# ASSOCIATION BETWEEN POLYPHARMACY AND Α-KLOTHO IN US OLDER ADULTS: THE MEDIATION ROLE OF INFLAMMATORY MARKERS

**DOI:** 10.1093/geroni/igae098.2284

**Published:** 2024-12-31

**Authors:** Kai Wei, Chun Chen, Yanping Yang, Libin Wang, Shuimei Sun

**Affiliations:** Guizhou Provincial People’s Hospital, Guiyang, Guizhou, China (People’s Republic); Guizhou Provincial People’s Hospital, Guiyang, Guizhou, China (People’s Republic); Guizhou Provincial People’s Hospital, Guiyang, Guizhou, China (People’s Republic); Guizhou Provincial People’s Hospital, Guiyang, Guizhou, China (People’s Republic); Guizhou Provincial People’s Hospital, Guiyang, Guizhou, China (People’s Republic)

## Abstract

**Introduction:**

Polypharmacy may lead to adverse health outcomes in older adults, but the relationship between polypharmacy and accelerated aging is not well understood. This study aims to investigate the association between polypharmacy and α-klotho, a blood anti-aging protein, using large population-based data.

**Methods:**

The study selected 3569 participants aged 65-79 years with serum α-klotho test data and prescription information from the National Health and Nutrition Examination Survey (2007-2016). Participants taking 5 or more medications simultaneously were classified as having polypharmacy. Weighted linear regression analyses were conducted to evaluate the association between polypharmacy and α-klotho. Mediation analysis was conducted to investigate the impact of inflammatory biomarkers, including the systemic immune-inflammation index, systemic inflammation response index, and neutrophil-to-lymphocyte ratio, on the relationship between polypharmacy and α-klotho levels.

**Results:**

The polypharmacy group consisted of 1,591 participants (weighted percentages 42.7%). The median level of α-klotho in the polypharmacy group was 733.16[594.47, 911.63] pg/mL, which was significantly lower than that in the non-polypharmacy group. After adjusting for multiple variables, polypharmacy remained significantly negatively associated with α-klotho (β-coefficient -0.0506, 95% CI -0.0855 to -0.0157), and this significant association were observed in men and women, respectively. Further mediation analyses revealed that the association between polypharmacy and α-klotho was mediated by inflammatory biomarkers, with the proportion of mediation ranging from 6.11% to 11.82% (all P < 0.01).

**Conclusion:**

These findings suggest that polypharmacy is closely linked to circulating α-klotho levels, which may be partly mediated by inflammation.

